# Stuffing Teeth

**Published:** 1866-09

**Authors:** 


### STUFFING TEETH.

*Editor Register:*—In the March No. of the REGISTER appeared an *Editorial* on Stuffing Teeth, and in the July No. appeared a communication on the same subject. I was highly pleased with the *Editorial*, and cannot find fault with a part of the communication. I suppose that the reason I cannot find any fault with a portion of the communication is, that the part to which I allude was not written at all. "B," in his communication has just affirmed a thing without showing a single cause for his assertions; besides, he is too sweeping in his remarks; so much so, that some of these "old time operators" are very much incensed. The idea conveyed, if it is not expressed, is that all who fill teeth the old way are stuffers, in the sense of the caption. These very men to whom I have reference are of the very best class of operators; and if all the miserable stuffing of teeth were left for such operators as they are, my word for it, there would be little fault found with the dental profession for poor operations.

I am fully aware that some of these old practitioners of dentistry make the most miserable operations; but to say that many of those old style operators, and leave the reader to infer that they alone are doing all the "stuffing" of the teeth, is injustice to both classes of operators.

I am fully impressed with the belief that this is a good way to stir up this class of operators, to do their duty to themselves, and to the profession. And in doing so, if the worst comes to the worst, call names. I am sure that some men would blush to have their poor operations held up to the world as such. I know I should. But to the point.

The heading of this article implies that some body is filling teeth in such a manner that they will not stand the test. That is all very true. I will state what in my opinion is a poor filling. In the first place, a soft filling (I mean gold) is of very little account to the wearer. A filling that is not sufficiently hard to resist the secretions of the mouth, will be of very little service to the tooth. Some of the tests to which a filling may be submitted are, First: is it hard and firm? Secondly: does it fit up closely to every part of the wall of the cavity? Thirdly: does it stand out flush with every part of the cavity? Fourthly: is it solid from the bottom of the cavity, and if in an approximal cavity, does it fit up tight to the margin of the cavity next to the gum? If you find a filling so soft that to apply the flat side of a burnisher to it, you can settle it down, and in many cases mash out a creamy looking substance, you are safe in saying that that plug is of no account. I think such a filling had better not be there; the tooth in my opinion would be better without anything at all in it. Then again, a filling may be hard in the middle, and not fit close to the walls of the cavity, and be of as little benefit as though it were soft all through. About the time of the appearance of the March No. of the REGISTER, a young lady presented herself to have some teeth filled. On examination I found she had seven cavities that had been filled, three of which had fallen out. Of all the stuffing teeth that I had ever seen or heard of, I think this was the worst. This work had not been done by one of these old time dentists, but by a live young Indiana dentist.

I have heard the word "supremely ridiculous;" but I think this case was "supremely miserable butchery." An excavator might be run to the bottom of any of the fillings with ease. Had I the choice in the matter, I would much prefer the gold out of the teeth than in them. I removed the fillings, and found that he had not cleansed out the cavities at all, but had just scraped off a little of the out side of the decay and left the balance.

Some time since another young lady presented herself for the filling of some teeth, who had been in the hands of one of our old time operators. This was about the same as the first case, with one exception. He had used three kinds of materials, gold, amalgum, and os-artificial. The gold fillings were a spongy mass of stinking gold. The oxi-chloride of zinc was nearly all washed out, while the amalgam was black as charcoal, and really the best filling she had. In this, like the other case, the cavities had been poorly prepared.

Still one more case, and I am done. A gentleman who is abundantly able to pay for good operations, had been in the hands of two city dentists, (both graduates.) He wanted something done to his teeth. I found them in a very bad condition indeed. They were so nearly like both the above cases I have just mentioned that it is hardly necessary to particularize. He had gold and os-artificial in his teeth, neither of which was of any benefit to them.

As the mallet seems to be the only instrument that some men can fill a tooth with, I design a word on that instrument. I don't mean to cast a slur on those who use the mallet exclusively; by no means. I am one of that kind myself, in as far as I can use it. There are some teeth that I must confess I cannot fill with the mallet. I know some of our best operators who use the mallet altogether; but there are men who boast of their success with the mallet; and yet I doubt seriously whether they can fill a difficult cavity well with any kind of instrument. Yet they condemn all those who differ with their notions of things.

The mallet in the hands of those who use it, is probably the best instrument for filling they can use. I think I can fill a tooth better with the mallet than by any other process. While I think it the most efficient in may hands, I know of dentists who never use the mallet or adhesive foil, that can fill a tooth as well without it as I can with it. And in one class of cavities, and one particular condition of the mouth, I think they can beat me. That cavity is an approximal inferior molar or bicuspid cavity. In the condition is a profuse discharge of saliva.

To fill a tooth well with the mallet requires considerable practice, as well as a correct understanding of its use. Some men, I am persuaded, fail from want of a proper knowledge of how to use it, and not having a sufficient understanding of its application, fail; and because they do fail, they are ready to condemn all who use it, and assert that their fillings will ultimately fail also. The difference in the species of operators is the same as in everything else. Some men can never make a good mallet filling; others again will never make good soft foil fillers. Some men are naturally good blacksmiths, others are naturally good carpenters and joiners. Now no one would expect the man that is well adapted to the trade of blacksmithing to make a good wood workman. So it is with dentistry. Some can make a better filling of soft foil than they can of any other material. Others make better of adhesive foil than they can of anything else: and there are still others who cannot make good fillings of anything except amalgams. And I believe that a great many men have missed their forte entirely, by attempting to practice dentistry. But to those who are determined to practice dentistry I would say, find out how you can fill a tooth best, and leave others to do the same, and for the sake of harmony in the profession, let others alone. I don't mean by this that we will not meet in our societies, and discuss these things; but that we will not accuse our neighbor as being a quack, because he fills teeth different from us. If I had not met in these dental meetings, I presume I should be still trying to fill teeth the old way, (as it is called.) I hear of a new method, differing widely from any thing I have ever seen. It is the squirting process. I heard a dentist say, a few days ago, that he could take a "*squirt gun*" and squirt as good a gold filling into a tooth as some have, who come to his office.

I have already strung out this to very much greater length than I intended. So I will close by naming one man who never filled a tooth with the mallet in his life; and although now dead, yet there are thousands of fillings made with soft foil that have already stood for forty years, and will still stand as monuments to his memory as long as the patients live. I mean Dr. Badger.

Poor filling attaches to the operator, rather than the manner of the operation.

